# An estimate of diapycnal nutrient fluxes to the euphotic zone in the Florida Straits

**DOI:** 10.1038/s41598-017-15853-0

**Published:** 2017-11-23

**Authors:** Jia-Zhong Zhang, Molly O. Baringer, Charles J. Fischer, James A. Hooper V.

**Affiliations:** 1 NOAA, Ocean Chemistry and Ecosystems Division, Atlantic Oceanographic and Meteorological Laboratory, Miami, FL 33149 USA; 2 NOAA, Physical Oceanography Division, Atlantic Oceanographic and Meteorological Laboratory, Miami, FL 33149 USA; 3 University of Miami, Cooperative Institute for Marine and Atmospheric Studies, Miami, FL 33149 USA

## Abstract

A recent hydrographic survey of the Florida Current at 27°N revealed an enhanced upward flux of nutrients along the Florida coast. Geostrophic flow of the Gulf Stream through the narrow Florida Straits causes an uplift of the nutricline toward its western edge, shoaling the mixed layers into the base of the euphotic zone. At a nearshore station, nitrate, phosphate, and silicate concentrations reached 19, 1.4, and 10 µM, respectively, at a water depth of 27 m. Furthermore, nutrient vertical gradients below the mixed layer increased with decreasing seafloor depth toward the Florida coast. The estimated vertical eddy diffusive nutrient fluxes across diapycnal surfaces reached 0.40–83.7, 0.03–6.24, and 0.24–45.5 mmol m^−2^ d^−1^ for nitrate, phosphate, and silicate, respectively, along the shore. Estimated fluxes span a wide range due to the range of diffusivity measured. The lower end of estimated fluxes are comparable to open ocean values, but higher end of estimates are two orders of magnitude greater than those observed in open ocean. The diapycnal nutrient fluxes declined rapidly offshore as a result of decreasing vertical gradients of nutrient concentration.

## Introduction

Nutrient transport processes to the euphotic zone of the ocean surface are essential for oceanic primary production and biological carbon sequestration. Phytoplankton production consumes nutrients at the sunlit surface. The resulting particulate organic matter sinks and then decays below the mixed layer, regenerating nutrients in the thermocline. A resupply of nutrients from the thermocline below is needed to sustain primary production at the surface. The diapycnal supply of nutrients to surface waters follows Fick’s law in which the upward flux of nutrients is a product of diapycnal diffusivity and the vertical nutrient concentration gradient as follows,

$${\rm{F}}={k}_{v}(\mathrm{dN}/\mathrm{dz})$$

where F is the upward nutrient flux, *k*_*v*_ is the diapycnal diffusivity, and dN/dz is the nutrient vertical concentration gradient. Although increasing vertical nutrient concentrations with depth can drive the diffusion of nutrients upward to the surface, molecular diffusion itself is too slow to reduce the existing concentration gradients. Steep gradients are largely maintained by a stratification of the upper water column due to density gradients.

In the ocean, physical disturbances by waves and wind result in vertical mixing in the water column that becomes a major mechanism for supplying nutrients to the mixed layer. However, measurements of vertical mixing in the main thermocline are about an order of magnitude smaller than what is required to sustain a downward flux of heat and buoyancy to close the global ocean’s overturning circulation^1^. Therefore, other processes must be operating at smaller spatial and temporal scales, such as enhanced mixing in boundary currents and over rough topography^2,3^. At high latitudes, winter convective mixing has been considered a dominant process in replenishing nutrients to the surface. In tropical and subtropical oceans, wind-driven upwelling, advection along density surfaces, storms, fronts, and eddy pumping have all been examined as processes supplying nutrients to the euphotic zone^4–11^.

The Gulf Stream has been characterized as a nutrient stream that irrigates the surface of the North Atlantic Ocean^12–16^. However, the surface waters of the Gulf Stream are oligotrophic (i.e., they contain the lowest concentration of macronutrients in oceanic waters) and, as such, have been used as a background matrix for low-level nutrient measurements (so called “low nutrient seawater”)^17,18^. In fact, the core of the Gulf Stream flows along the western edge of the Florida Straits, with its maximum velocity at the ocean surface, while the core of the nutrient stream is about 500 m below the surface and slightly offshore of the high-velocity core of the Gulf Stream^12^. As the Gulf Stream flows north through the Florida Straits, its surface waters spread into the subtropical gyre, and its nutrient-rich, upper-thermocline waters penetrate farther north into the subpolar gyre^12–16,19–23^. During this extension of the Gulf Stream flow, the original subsurface water masses and their denser isopycnals progressively outcrop at higher latitudes, with nutrients reaching the surface euphotic zone and supporting high primary production in the North Atlantic subpolar gyre^12–16,19–23^.

In addition to the along-stream northward transport of nutrients, there is also an across-stream westward transport of nutrients induced by the geostrophic flow of the Gulf Stream. Redfield speculated that the fertility of the waters along the Atlantic coast of North America is largely dependent on the transport of nutrients from open ocean basins across the Gulf Stream to west of the current system^24^. Summer upwelling and the intrusion of high nutrient, high salinity, and low temperature waters from the deeper Gulf Stream have been observed along the northeast coast of Florida, the Mid-Atlantic Bight, and the Florida Keys^6,25–29^. Early studies focused primarily on the area from northern Florida to Cape Hatteras, where there is a relatively broad continental shelf^6,25–28^. Upstream of the Gulf Stream system, the Florida Current is confined to the narrow Florida Straits, with the maximum northward current velocity reaching 2 m s^−1^ near the surface. The Florida Current has been continuously monitored since the early 1980s as the most important component of the Atlantic Meridional Overturning Circulation in support of global climate variability studies^30,31^.

While tilted isopycnals are well known in the Florida Current system from previous hydrographic surveys, to our best knowledge, strong nutrient vertical gradients in shallow nearshore waters have not been documented. During a recent cruise in which shallow coastal stations were occupied for the first time, we observed high nutrient concentrations in the lower euphotic zone of surface waters at the western edge of the Florida Current. We also observed steep vertical nutrient gradients below the mixed layer in these shallow waters. Nutrient vertical profiles, combined with previously determined eddy diffusivity values from Multi-Scale Profiler measurements in the Florida Straits, enabled us to estimate the vertical nutrient flux in the Florida Current.

## Methods

**Figure 1:**
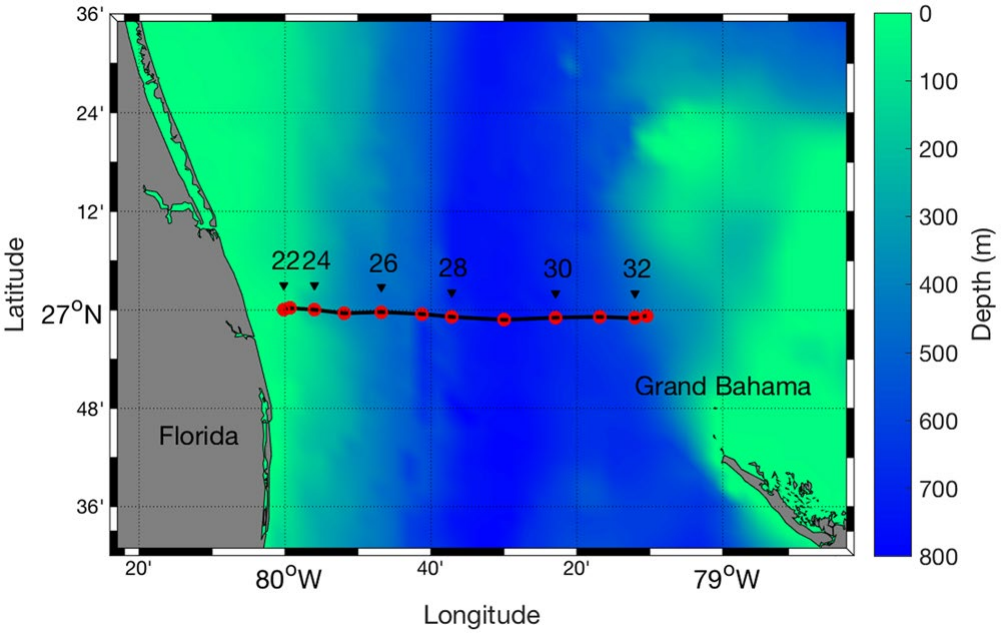
Station location at 27°N and the bathymetry of the Florida Straits (generated by M-Map v1.4 h, https://www.eoas.ubc.ca/~rich/map.html and MATLAB v2016a, https://www.mathworks.com/).

Nutrient data were collected on board the NOAA Ship *Ronald H. Brown* during the second Gulf of Mexico and East Coast Carbon (GOMECC-2) cruise from July 21 to August 13, 2012. The objective of the cruise was to monitor the long term ocean acidification trends along the eastern coastal ocean on interannual time scales. Full water column measurements of carbon, nutrients, and other relevant physical, chemical, and biological parameters were gathered on a series of hydrographic transects^32^. A hydrographic section across the Florida Current was one of eight cross-shelf transects occupied during the cruise. A total of 12 stations, i.e., stations 22–33 (Fig. 1), were occupied across the Florida Straits along 27°N from July 30, 2012, 1900 GMT to July 31, 2012, 1900 GMT.

During the cruise, the ship conducted nearly continuous operations of Bathy 2000 3.5 kHz and Seabird 12 kHz depth estimations recorded in the SCS system. Full water column sampling was performed with a CTD/Rosette system containing 24 10-liter Niskin-type bottles. The CTD package consisted of a Sea-Bird Electronics (SBE) 9 plus CTD with dual pumps, dual temperature (SBE3), conductivity (SBE4), and dissolved oxygen (SBE43) sensors and a Simrad 807 altimeter. A single Guildline Autosal model 8400B salinometer was used for shipboard measurements of salinity from discrete Niskin bottle samples. The water column was sampled at depth intervals of 5 m at near surface to 50 m at depth with the deepest samples taken within 5–10 m of the seafloor. Surface mixed layers were defined as the depth where the density gradient with depth was greater than 0.004 kg m^−3^ m^−1^ ^33^.

Details on the parameters sampled can be found in a report prepared after the cruise^34^. The sampling and analysis protocols for discrete samples closely followed the procedures of the GO-SHIP Repeat Hydrography Program^35^. Dissolved nitrate, phosphate, and silicate were sampled in 30-ml, acid-cleaned plastic bottles after three sample rinses and measured with a gas-segmented continuous flow auto-analyzer (AA3, SEAL Analytical, Inc.) on board the ship after the samples had warmed to room temperature. Samples for nitrate analysis were passed through a copper-coated cadmium column^36^, which reduced nitrate to nitrite. The resulting nitrite concentration (i.e., the sum of nitrate + nitrite) was then determined by diazotizing with sulfanilamide and coupling with N-1 naphthyl ethylenediamine dihydrochloride to form a pink azo dye, absorbance of which was measured at 540 nm. Phosphate in the sample was determined by its reaction with molybdate acid to form a phosphomolybdate complex that was subsequently reduced by hydrazine to form phosphomolybdenum blue, the absorbance of which was measured at 736 nm^37^. Silicate was analyzed by reacting dissolved silicate in the sample with ammonium molybdate in an acidic solution to form β-molybdosilicic acid, which was then reduced by ascorbic acid to form molybdenum blue. The absorbance of the molybdenum blue was measured at 660 nm. Oxalic acid was added to prevent a reduction of excess molybdate and to minimize the interference of phosphate in the sample^38^.

A mixed stock standard solution consisting of nitrate, phosphate, and silicate was prepared by dissolving high purity standard materials (KNO_3_, KH_2_PO_4_, and Na_2_SiF_6_) in deionized water. Working standards used to calibrate the auto-analyzer were prepared fresh daily by diluting the stock solution in low nutrient seawater and referenced against the nutrient Certified Reference Materials provided by the Meteorological Research Institute of Japan.

## Results and Discussion

**Figure 2:**
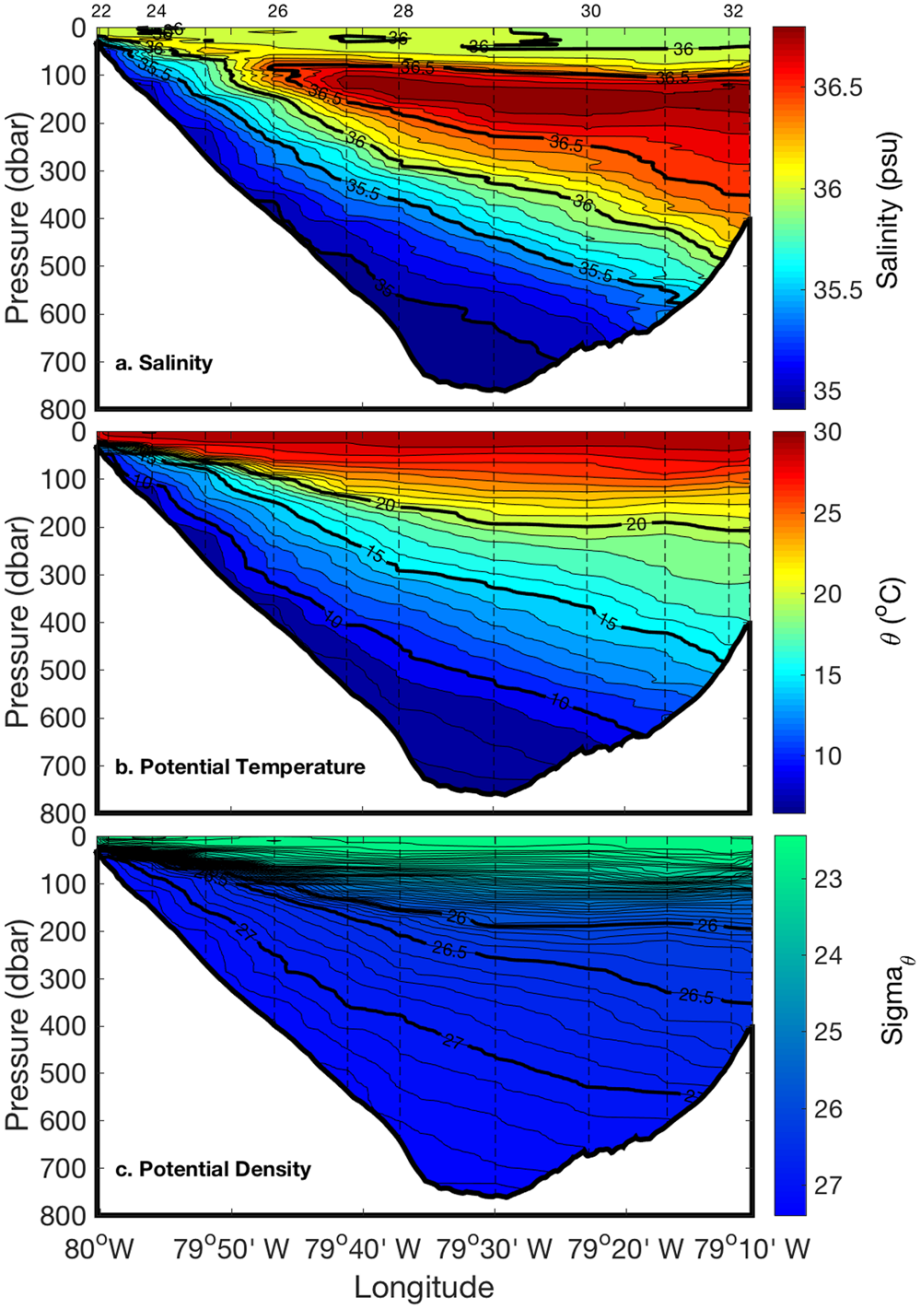
Sections of salinity (**a**), potential temperature (**b**), and potential density (**c**) of the Florida Current along 27°N in the Florida Straits. Station numbers are shown at the top of each panel. Bathymetry is based on shipboard measurements. Section plots were created by M-Map v1.4 h with a linear interpolation gridding method (https://www.eoas.ubc.ca/~rich/map.html and MATLAB v2016a, https://www.mathworks.com/).

Sections of measured salinity, temperature, and the calculated potential density reference to the sea surface are shown in Fig. 2. The top 50 m of the Florida Current consists of relatively low salinity water (around 36). The maximum salinity water (>36.5) sits at mid depth and forms a wedge-shaped section, indicating an offshore origin of Central Atlantic Water. The lowest salinity water (35–35.5) is located at the bottom of the section, which originates from low salinity-high nutrient Antarctic Intermediate Water^39^. Although there is a monotonic decrease in temperature with depth, the vertical temperature gradient progressively increases with decreasing seafloor depth, reaching a maximum at the western edge of the Florida Straits. The intrusion of low temperature-low salinity water originating from Antarctic Intermediate Water to the shallow coast is apparent in Fig. 2. The density section closely resembles the temperature section. Due to the geostrophic flow of the western boundary current system, the density surface is uplifted toward the western coastal shoreline. The density surface of σ_ѳ_ = 27.0 is about 550 m deep at the eastern edge of the Florida Current but shoals to 60 m at the western edge. Likewise, an observed lighter density surface of σ_ѳ_ = 26.5 is about 350 m deep at the eastern edge and is lifted to a depth of 30 m at the western edge, well into the euphotic zone.

**Figure 3:**
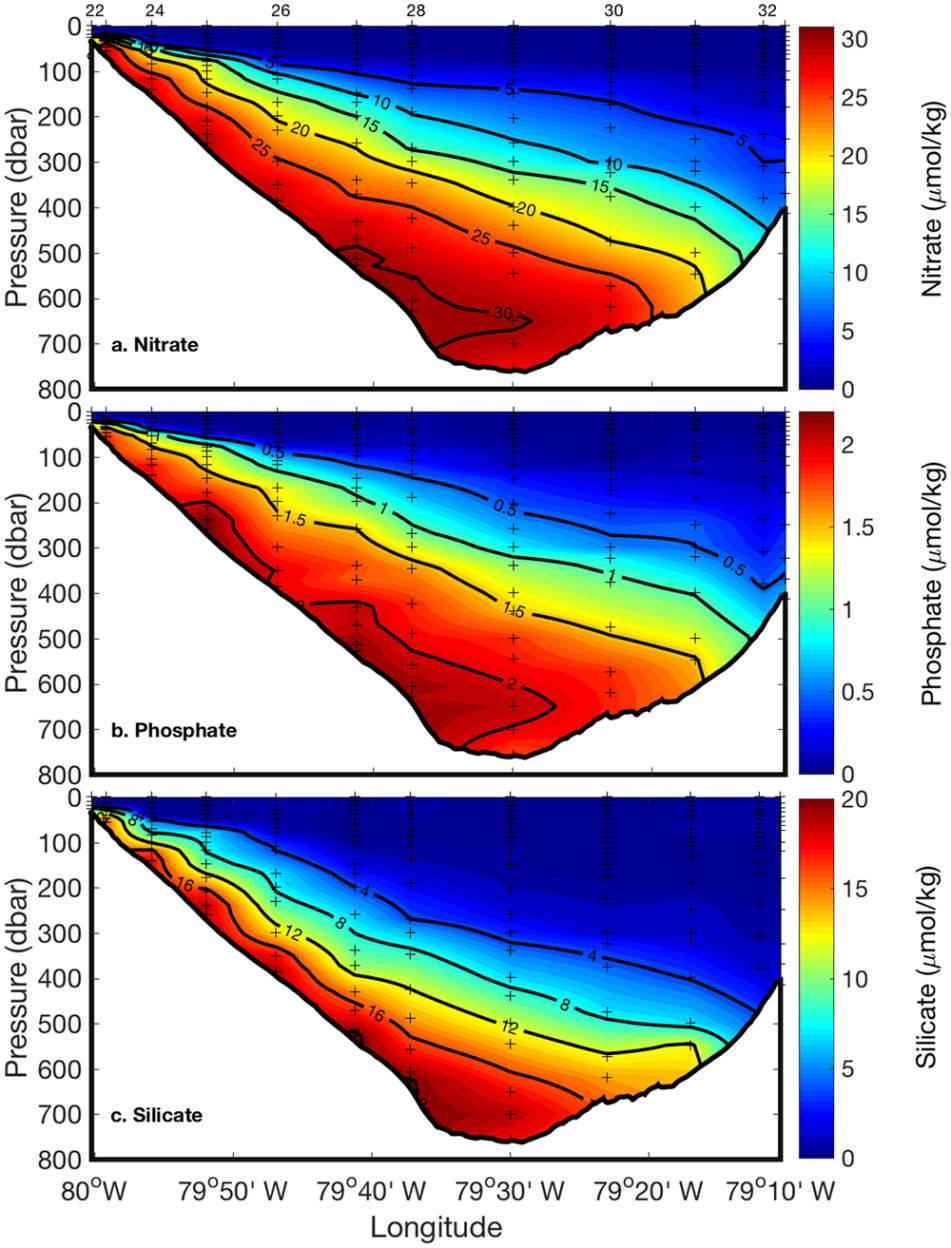
Sections of nitrate (**a**), phosphate (**b**), and silicate (**c**) of the Florida Current along 27°N in the Florida Straits. Station numbers are shown at the top of each panel, and sampling locations are indicated with crosses in each panel. Section plots were created by M-Map v1.4 h with a linear interpolation gridding method.

**Figure 4:**
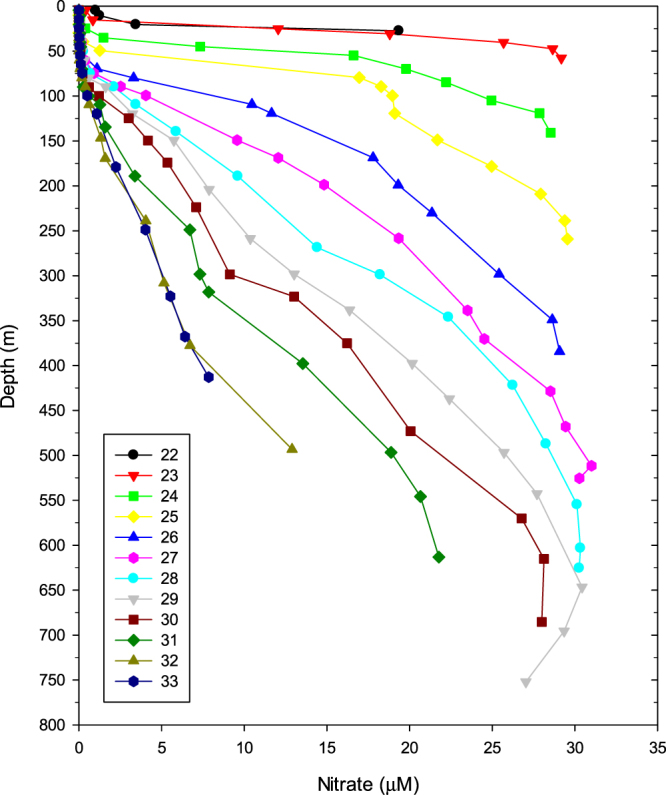
Vertical profiles of nitrate at stations along the 27°N transect (stations 22–33 as shown in Fig. 1) in the Florida Straits. The symbols indicate the depth at which samples were taken at a given station. Note an increasing nitrate gradient below the mixed layer with decreasing water depths toward the western coast. The station bottom depth (see Table 1) is approximately 10 m below the deepest sample taken at stations deeper than 100 m and 5 m at stations shallower than 100 m.

Sections of nitrate, phosphate, and silicate measured during the cruise are shown in Figs 3 and 4. The overall structure of the nutrient sections resembles that of density. Low nutrient concentrations occur in the warm surface layer. The thickness of the low nutrient surface layer decreases from 200 m at the eastern edge to less than 20 m at nearshore stations along the western edge (see Fig. 4 for nitrate profiles). Although all nutrient concentrations increased with depth and reached their maximum concentration at the bottom of the Florida Straits, silicate differed from phosphate and nitrate. Phosphate profiles are analogous to those of nitrate, as their concentrations follow the Redfield Ratio in deep waters (nitrate:phosphate ratio of 14.8 ± 2.3). Silicate concentrations are depleted deeper than nitrate and phosphate, with <4 µM at 400 m in the central to eastern Straits of Florida due to the slower rate of dissolution of opal to dissolved silicate compared to the rate of microbial decomposition of organic matter to regenerate nitrate and phosphate^40^. The silicate to nitrate ratios in the Florida Current increased with water depth, ranging from <0.1 in the upper thermocline to about 0.6 in bottom waters.

Bottom waters originating from Antarctic Intermediate Water are rich in nutrients as a result of the remineralization of organic matter along their northward flow^41^. Owing to the geostrophic flow of the western boundary current system in the Florida Straits, the tilted density surfaces also uplift the nutricline surface toward the western coastal shoreline. As a result of the thinning mixed layer, nutrient-rich thermocline waters are closer to the sea surface at the western edge. At the shallowest station, i.e., station 22, nitrate concentrations reached 1 µM at a water depth of 4.6 m, in contrast to undetectable nitrate levels in the surface waters of the central Straits of Florida. At this station, nutrient concentrations increased rapidly with depth, e.g., nitrate and phosphate concentrations reached 19 µM and 2 µM, respectively, at a water depth of 27 m, which is well within the euphotic zone of the region^42^.

Furthermore, as the nutriclines were lifted and compressed towards the western coast, vertical nutrient gradients progressively increased with decreasing seafloor depth, reaching a maximum at the western edge of the Florida Straits (Fig. 4). At station 29, the deepest station in the middle of the Straits (780 m), the nutrient gradient below the mixed layer (100 m) is 0.08 and 0.0066 µM m^−1^ for nitrate and phosphate, respectively. In contrast, at station 22, the shallowest station at the western edge of the Florida Straits (42 m), the nutrient gradient below the mixed layer (20 m at this station) increased to 2.3 and 0.172 µM m^−1^ for nitrate and phosphate, respectively. Across the Florida Straits, the vertical nutrient concentration gradients below the mixed layer increased 28- and 26-fold for nitrate and phosphate, respectively.

According to Fick’s law, a greater nutrient concentration gradient below the mixed layer results in a greater upward nutrient flux in the eddy diffusion processes. Vertical diffusivity in the Florida Straits was determined by Winkel *et al*.,^43^ who used a Multi-Scale Profiler to collect comprehensive measurements of diffusivity at seven stations across the Florida Straits. Their study identified five mixing regimes in this vertically sheared environment. While moderate mixing was observed in the most interior portion of the Florida Straits, with diffusivities ranging from 0.07 to 0.7 cm^2 ^s^−1^, strong mixing was found in the turbulent stratified boundary layers, particularly within 100 m of the channel floor, where diffusivities averaged 4.2 cm^2 ^s^−1^. While microprofilers have successfully measured eddy diffusivity in shallow marine environments^44^, no measurements were gathered in the shallow coastal waters of the Florida Current by Winkel *et al*.^43^. Considering the wide range of measured diffusivities and uncertainty due to sampling gaps in coastal shallow waters, we adapted their entire range of measured eddy diffusivity values of 0.02–4.2 cm^2 ^s^−1^ in the Florida Current for our nutrient diapycnal flux calculation.

Using the range of eddy diffusivity reported by Winkel *et al*.^43^ for the Florida Straits and measured nitrate, phosphate, and silicate gradients below the mixed layer at the given stations, we calculated the upward diapycnal nutrient fluxes across the Florida Current (Table 1). A striking feature is the strong gradient in diapycnal nutrient fluxes across the Florida Straits. At the western edge of the Florida Current, nitrate, phosphate, and silicate fluxes reach 0.4 to 83.7, 0.03 to 6.24, and 0.2 to 45.5 mmol m^−2^ d^−1^, respectively. The vertical nutrient fluxes, however, decline rapidly offshore. Nitrate, phosphate, and silicate fluxes are 0.2 to 3, 0.001 to 0.15 and below 0.1 mmol m^−2^ d^−1^, respectively, in the central Straits of Florida.

**Table 1:** Vertical nutrient concentration gradients below the mixed layer and estimated vertical nutrient fluxes along the transect at 27°N in the Florida Straits. A range of flux estimates were calculated from the range of eddy diffusivities (*k*_v_) of 0.02–4.2 cm^2 ^s^−1^ measured by Winkel *et al*.^43^ in the Florida Straits.

station	longitude (°E)	bottom depth (m)	Vertical Gradient (μM m^−1^)			Nutrient Flux (mmol m^−2^ d^−1^)		
			NO_3_	PO_4_	Si(OH)_4_	NO_3_	PO_4_	Si(OH)_4_
22	80.003	42	2.307	0.172	1.254	0.40–83.7	0.030–6.24	0.22–45.5
23	79.986	67	1.228	0.092	0.584	0.21–44.6	0.015–3.33	0.10–21.2
24	79.931	159	0.956	0.061	0.406	0.17–34.7	0.010–2.19	0.07–14.7
25	79.865	276	0.525	0.036	0.239	0.09–19.1	0.006–1.31	0.04–8.7
26	79.781	404	0.243	0.017	0.079	0.04–8.8	0.003–0.61	0.01–2.9
27	79.686	554	0.154	0.007	0.019	0.03–5.6	0.001–0.27	0.003–0.69
28	79.620	653	0.096	0.007	0.006	0.02–3.5	0.001–0.25	0.001–0.20
29	79.499	780	0.106	0.004	0.002	0.02–3.8	0.001–0.14	0.001–0.07
30	79.383	700	0.072	0.004	0.001	0.01–2.6	0.001–0.15	0.001–0.04
31	79.282	628	0.071	0.004	0.000	0.01–2.6	0.001–0.16	0.001–0.18
32	79.200	509	0.011	0.001	0.000	0.002–0.4	0.0002–0.05	0.000
33	79.174	431	0.029	0.001	0.000	0.005–1.0	0.0001–0.03	0.000

Pelegri and Csanady (1991) analyzed data from several hydrographic sections to examine nutrient transport along the Gulf Stream^12^. They used a two-box model to separate upward entrainment from two-way exchange and estimated the diapycnal mixing between the upper thermocline and surface layers. They found a two-way mean exchange of 2.4 Sv between the Florida Straits and a section at 36°N, which produced an upward net transfer of 37000 mol s^−1^ of nitrate^13^. Their nitrate flux estimate, averaged over 1500 km of the Gulf Stream between these two sections, is of the same order of magnitude as our estimate in the Florida Straits, although the two-box model, dealing with large spatial scale processes, cannot resolve across-stream variability in the vertical nutrient gradients of the Florida Straits. Palter and Lozier (2008) subsequently chose relevant diffusivity values for different parts of the Gulf Stream^15^. In the Florida Current, they adopted the entire range of vertical eddy diffusivities measured by Winkel *et al*.^43^ (0.02–4.2 cm^−2^ s^−1^) and derived a phosphate flux divergence ranging from 10^−12^ to 10^−9^ mmol m^−3^ s^−1^. Considering the 100 m water depth used in their flux calculation for the Florida Current^15^, the high end of their flux estimate is similar to our calculated value in the central Straits of Florida. Our high flux values for shallow coastal waters are the result of strong nutrient vertical gradients that have not been documented in the historical hydrographic dataset and, therefore, were not used in previous flux estimates.

In the open Atlantic Ocean away from the Gulf Stream, upward fluxes of nitrate have been estimated to be 0.14 mmol m^−2^ d^−1^ in North Atlantic oligotrophic waters^45^. Eddy pumping by episodic storms has been observed to enhance upward nutrient fluxes from 1.24 to 4.15 mmol m^−2^ d^−1^ ^46,47^. Our estimated nutrient fluxes in the Florida Current span a wide range due to the range of diffusivity measured. The lower end of estimated fluxes are similar to open ocean values, but higher end of estimates are two orders of magnitude greater than those observed in oligotrophic open ocean gyre waters and 10 to 30 times greater than those observed in mesoscale eddies^45–47^.

The results presented in this study provide direct evidence in support of Redfield’s 1936 notion that the Gulf Stream is a major source of nutrients for North Atlantic coastal waters^24^. Our observations reveal that high nutrients reach the lower part of the euphotic zone in shallow coastal waters by the lifting of the nutricline on epipycnal surfaces. Concurrently, there is an enhanced diapycnal mixing of nutrients to surface waters driven by steep nutrient gradients below the shallow mixed layer. Along this 200-km long by 4.8-km wide stretch of the Florida coast, we estimated nutrient fluxes into the surface mixed layer from the Florida Current to be 2.6 × 10^5^ to 5.5 × 10^7^, 2.0 × 10^4^ to 4.1 × 10^6^, and 1.3 × 10^5^ to 2.8 × 10^7^ mol d^−1^ for nitrate, phosphate, and silicate, respectively.

Pelegri and Csanady (1991) showed that between the Florida Straits and Mid-Atlantic Bight the water flow doubled and nutrient transport trebles^12^. They calculated the Gulf Stream transports nitrate at a total rate of 309 kmol s^−1^ through the Florida Straits, with about 20% of this transport (62 kmol s^−1^) occurring on isopycnals with densities between 25.6–26.5 kg m^−3^. At 36°N, however, the total nitrate transport increases to 863 kmol s^−1^, in which a portion of the transport, 119 kmol s^−1^, occurs between densities of 25.6–26.5 kg m^−3^. They proposed that this nutrient transport convergence resulted from the entrainment of nutrient-rich thermocline waters into the Gulf Stream. Analysis of a more extensive dataset by Palter and Lozier (2008) has suggested that imported, extrasubtropical waters are the primary source of elevated nutrient concentrations in the nutrient stream^15^. Therefore, the source of nutrients in the Gulf Stream remains an open question. Our study provides evidence that the Florida Current might be an additional source of nutrients to the surface of the Gulf Stream.

Due to the dominant northward flow of the Gulf Stream, upward fluxes of nutrients are transported to the Mid-Atlantic Bight and farther north along the Gulf Stream into the North Atlantic gyre. There is also a southward flow of coastal waters observed along the Florida peninsula. Upward fluxes of nutrients in the Florida Straits might be transported by this counter coastal current and reach the Florida Reef Tract in the Florida Keys. Additionally, the lateral transport of nutrients across the Gulf Stream is likely to occur along Florida’s coast, although a different upwelling intensity might occur there due to the differing bottom topography and current intensity. Nutrient supply to the euphotic zone may also affect the eutrophication status of the shallow coral reef ecosystem along the Florida Reef Tract. Future studies to quantify the relative contributions of oceanic and terrestrial nutrients upon coral reef habitats are needed to adequately manage this valuable coastal ecosystem.
